# Rare Case of Persistent Descending Mesocolon Identified on Cross-Sectional Imaging

**DOI:** 10.7759/cureus.105915

**Published:** 2026-03-26

**Authors:** Anushika Maloo, Prashant Onkar, Suresh Phatak, Kajal Mitra, Rishi Meswani

**Affiliations:** 1 Department of Radiodiagnosis, NKP Salve Institute of Medical Sciences and Research Center, Nagpur, IND; 2 Department of Radiodiagnosis, Datta Meghe Institute of Medical Sciences, Wardha, IND; 3 Department of Radiodiagnosis and Imaging, NKP Salve Institute of Medical Sciences and Research Center, Nagpur, IND

**Keywords:** abdominal pain, computed tomography, congenital colonic anomaly, cross-sectional imaging, persistent descending mesocolon

## Abstract

Persistent descending mesocolon (PDM) is a congenital anatomical variant arising due to the failure of fusion of the descending mesocolon with the parietal peritoneum during fetal embryogenesis. In most individuals, this condition remains clinically silent and is detected incidentally on imaging or during abdominal surgery. However, its recognition is important because abnormal colonic mobility may predispose patients to future complications and may also influence surgical planning.

We present a case of an 18-year-old female who came to the hospital with recurrent pain in the abdomen and was further evaluated with abdominal ultrasound and contrast-enhanced computed tomography (CECT), where imaging findings led to the diagnosis of PDM. CT imaging enabled the accurate identification of this entity while excluding intestinal malrotation and other acute abdominal pathology.

## Introduction

During normal embryologic development, the descending colon becomes secondarily retroperitoneal after fusion of its mesentery with the posterior parietal peritoneum. Failure of this fusion results in persistence of the descending mesocolon, resulting in a mobile and medially displaced descending colon, a condition referred to as PDM [1].

PDM is rare and usually asymptomatic, which explains why it is often discovered incidentally during cross-sectional imaging or intraoperatively. However, its importance lies in the increased risk of complications such as volvulus, internal herniation, and technical difficulty during colorectal surgery. With the increasing use of cross-sectional imaging, especially CT in routine abdominal imaging, radiologists are frequently the first to identify this anomaly and therefore play a key role in its diagnosis.

## Case presentation

An 18-year-old female patient presented to the hospital with pain in the umbilical region for approximately 15 days, with a few episodes of vomiting, and a similar history in the past. On clinical examination, mild tenderness was noted in the umbilical region; however, no guarding or rigidity was present.

An initial abdominal ultrasonography examination did not reveal any abnormal findings. In view of persistent symptoms, a contrast-enhanced computed tomography (CECT) scan of the abdomen was performed for further evaluation.

**Figure 1:**
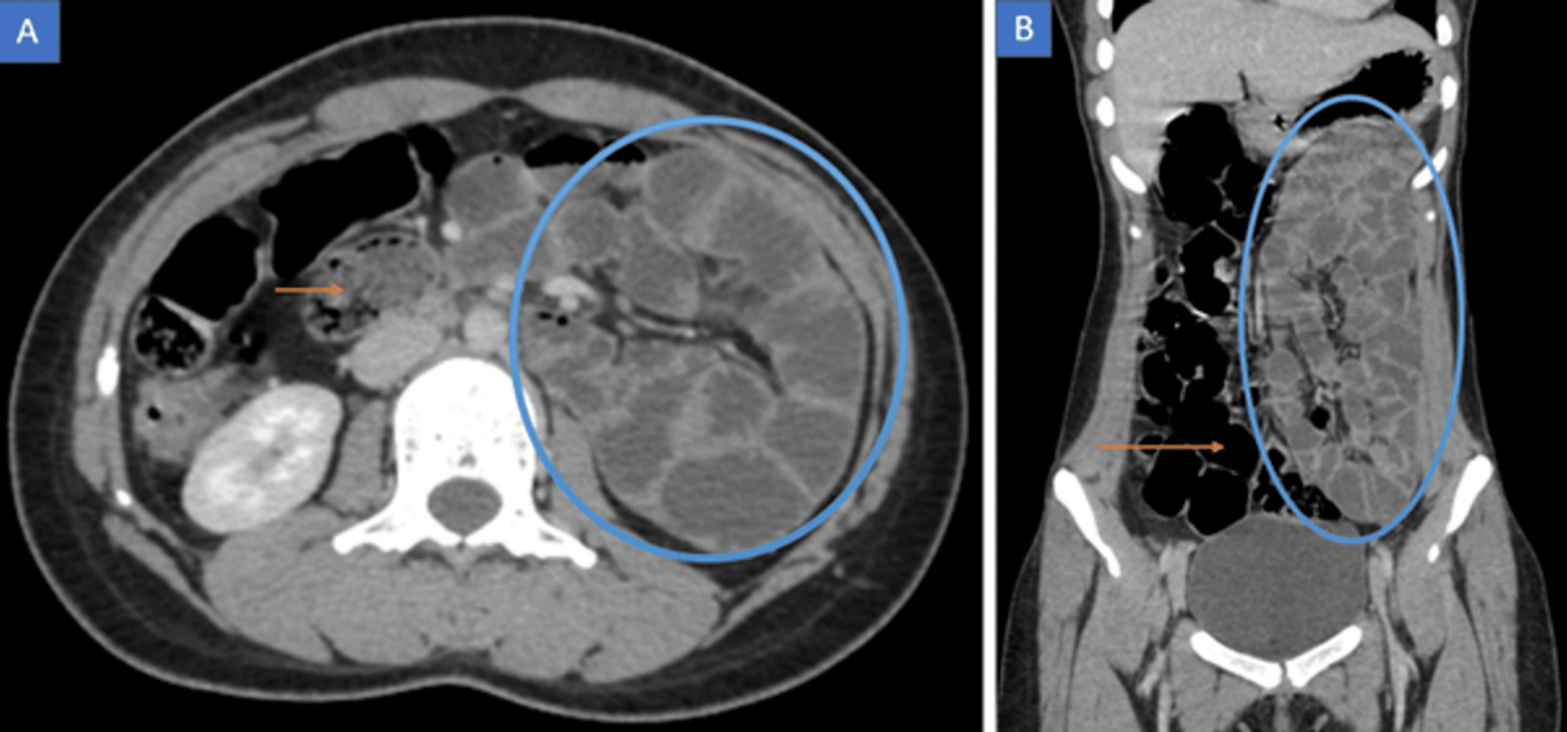
Contrast-enhanced axial and coronal CT images (porto-venous phase) (A) Contrast-enhanced axial CT abdomen image demonstrating medial displacement of the descending colon (orange arrow) with small bowel loops (blue circle) lateral to the colon occupying the left paracolic gutter. (B) Contrast-enhanced coronal CT abdomen image showing an oblique course and medialization of the descending colon (orange arrow) and lateralization of small bowel loops (blue circle).

**Figure 2:**
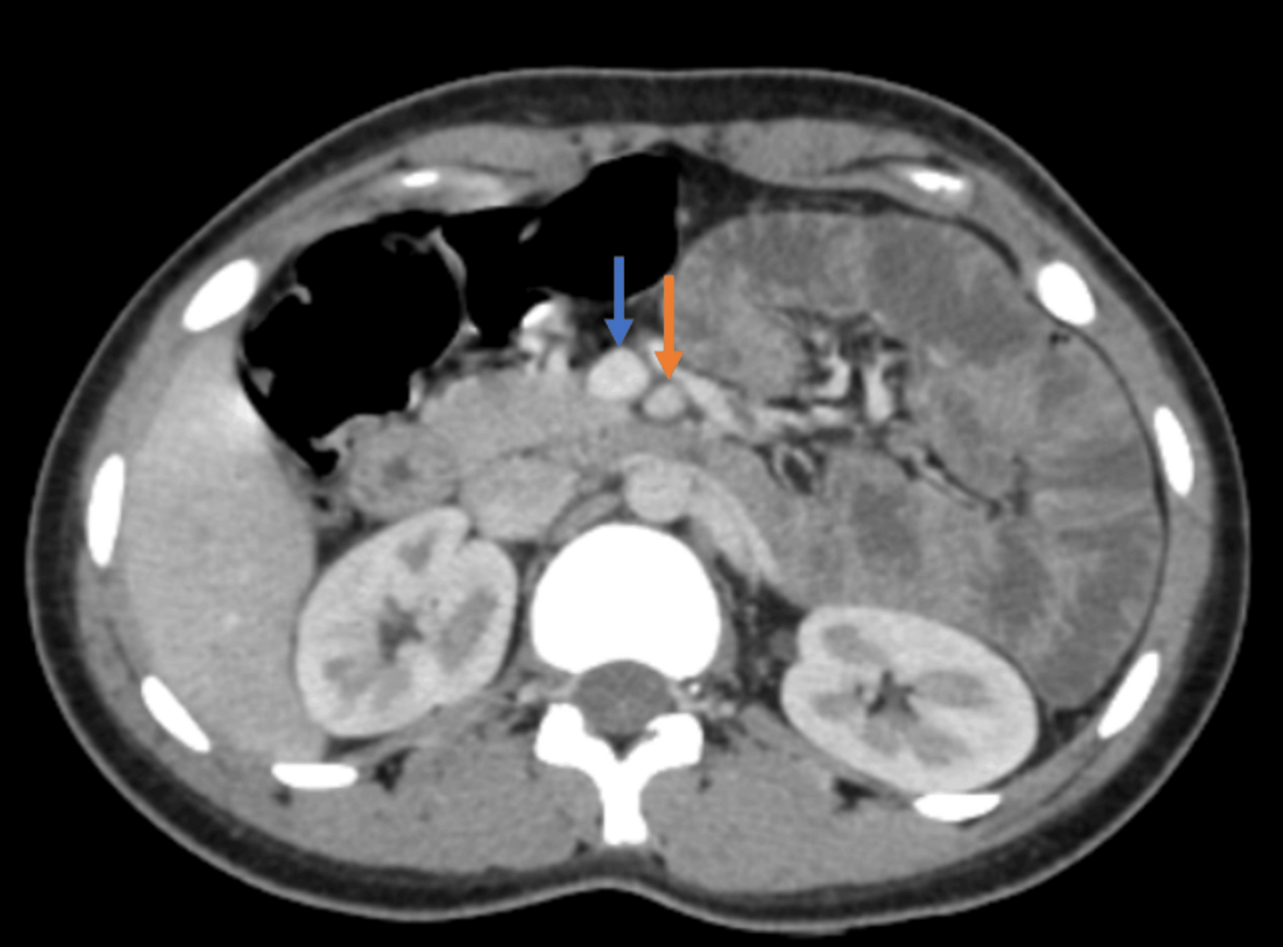
Contrast-enhanced axial CT abdomen (porto-venous phase) Contrast-enhanced axial CT abdomen image demonstrating a preserved superior mesenteric artery (orange arrow) and superior mesenteric vein (blue arrow) relationship.

The CECT scan demonstrated medialization and an oblique course of the descending colon. Small bowel loops were positioned lateral to the displaced descending colon, as shown in Figure 1. The ileocecal junction was normally located in the right iliac fossa. The bowel walls showed normal enhancement. No evidence of bowel obstruction or ischemia was noted. Superior mesenteric artery and vein relationship was maintained as shown in Figure 2. No signs of associated appendiceal inflammation were identified. No signs of any other abdominal organ pathology were detected. These findings were consistent with PDM.

No surgical intervention was needed, as there was no evidence of bowel obstruction, volvulus, ischemia, or other acute abdominal pathology on imaging. Therefore, the patient’s symptoms were considered nonspecific and not directly attributable to this anatomical variant. The finding of a PDM was incidental. The patient was managed conservatively with symptomatic treatment, including analgesics and antiemetics, and was discharged in a stable condition with advice for outpatient follow-up. The patient showed clinical improvement during the hospital stay, with resolution of abdominal pain and vomiting.

## Discussion

PDM is a congenital anatomical variant that results from the failure of fusion of the descending colonic mesentery with the posterior and lateral parietal peritoneum during embryogenesis [1]. As a result, the descending colon remains mobile and is displaced medially, leading to the absence of the colon from the left paracolic gutter and left iliac fossa. Most of the patients are usually asymptomatic, and the condition is often discovered incidentally during imaging or surgery. However, its clinical significance lies in its potential complications. Increased mobility of the colon predisposes patients to volvulus, internal herniation, and intussusception. Importantly, PDM represents a disorder of colonic fixation rather than a true intestinal malrotation, as normal midgut rotation and the superior mesenteric artery-vein relationship are typically preserved [2].

CT is the imaging modality of choice for diagnosis. Multiplanar CT allows accurate visualization of colonic position, length of mesocolon, vascular orientation, and associated complications. Although barium studies may demonstrate abnormal colonic positioning, CT provides a comprehensive anatomical assessment and is preferred [3].

Thus, identification and accurate reporting of PDM are essential, even when incidentally detected in asymptomatic patients, as they have significant implications for preventing potential complications and for planning future abdominal or colorectal surgical interventions [3].

Recent surgical literature has highlighted that the presence of a PDM may increase operative complexity, with higher rates of anastomotic complications and longer operative duration in colorectal procedures [4]. Additionally, altered vascular anatomy and atypical colonic fixation associated with this variant can pose technical challenges during laparoscopic colorectal surgeries, emphasizing the need for careful preoperative imaging assessment [5].

## Conclusions

Persistent descending mesocolon is a rare congenital anomaly that may be incidentally detected on cross-sectional imaging or present with nonspecific abdominal symptoms. Recognition of its characteristic CT features--medial displacement of the descending colon and lateral positioning of small bowel loops allows confident diagnosis. Proper identification and documentation of this entity can prevent diagnostic confusion, guide appropriate clinical management, and assist in surgical planning, thereby reducing the risk of future complications.
